# Role of GLD-3 in suppression of the germline stem cell fate.

**DOI:** 10.17912/micropub.biology.000259

**Published:** 2020-05-27

**Authors:** Ariz Mohammad, Jian Chen, Tim Schedl

**Affiliations:** 1 Department of Genetics, School of Medicine, Washington University in St. Louis, Missouri 63110.

**Figure 1:**
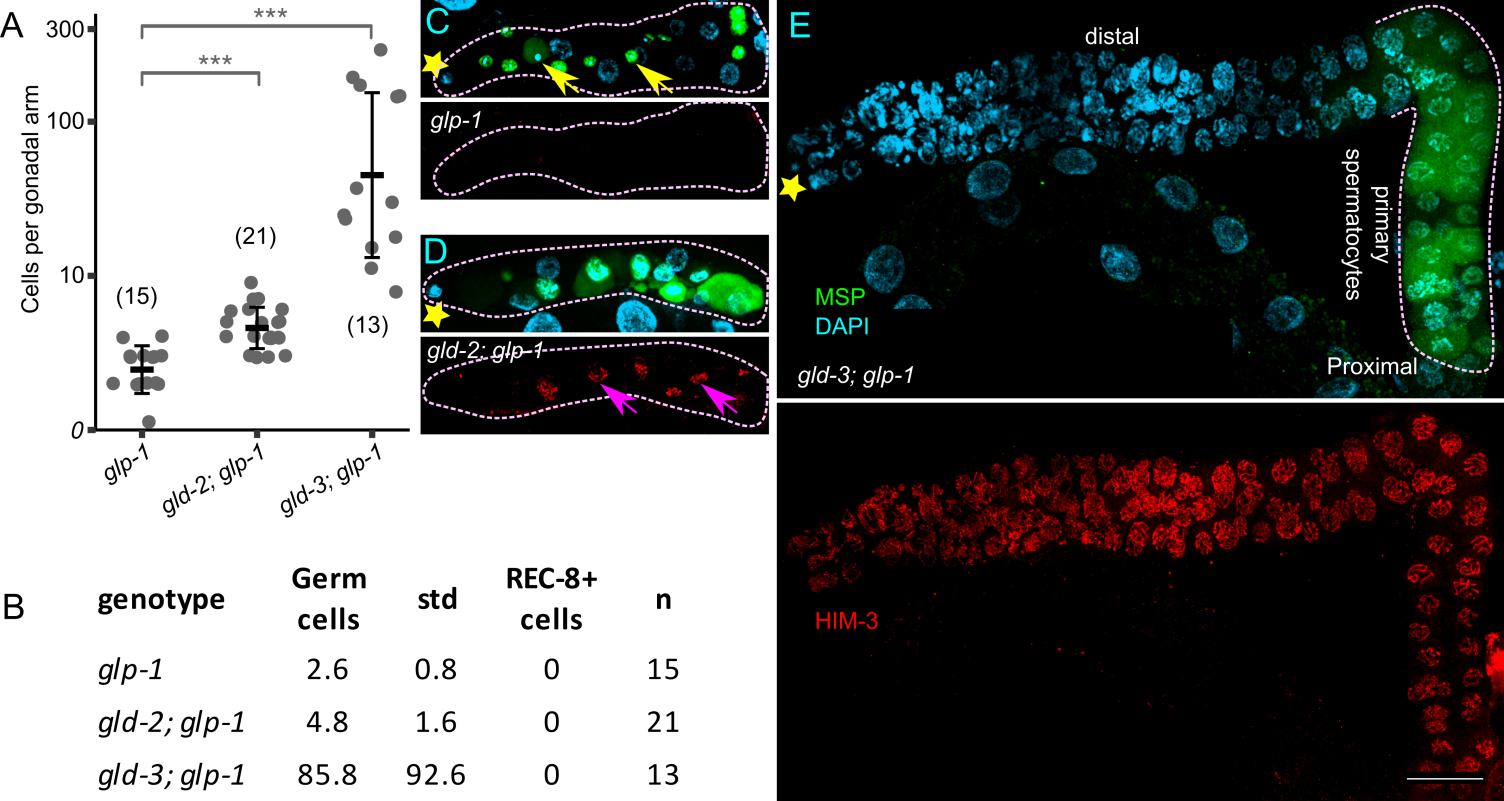
(A) Graph showing number of germ cells per gonadal arm of the indicated genotype. Number of germ cells were calculated as per: 1 germ cell = 4 sperm or = 2 secondary spermatocytes. Numbers represent n, *** represents p value of less than 0.0001 (two-tailed Student’s t-test), thick horizontal lines represent mean and vertical lines represent mean ± SD, note the logarithmic scaling of y-axis. (B) Table showing number of germ cells per gonadal arm in young adults. std, standard deviation. The average number of germ cells obtained for *glp-1* and *gld-2; glp-1* mutants is slightly lower than previously reported numbers (Francis *et al.*, 1995; Mohammad *et al.*, 2018), which were assessed in L3 stage germlines. This lower number could be due to germ cell/sperm loss during L4/adult stage. (C-E) Dissected young adult hermaphrodite germlines stained with anti-MSP antibody (green, top panels), DAPI to visualize the DNA (cyan, top panels) and anti-HIM-3 antibody (red, bottom panels). *, distal end of the gonad; yellow arrows, sperm; pink arrows, arrested late-pachytene/primary spermatocytes; scale bar: 25μm. In (E), large polyploid intestinal nuclei, below the germline, are visible by DAPI staining (top), but not visible by anti-HIM-3 staining (bottom). In *glp-1*, all the germ cells differentiate to sperm. In both *gld-2; glp-1* and *gld-3; glp-1* mutants, germ cells arrest as late-pachytene/primary spermatocytes, although in the latter some germ cells occasionally differentiate into sperm. The dotted line in E delineates arrested late-pachytene/primary spermatocytes in *gld-3; glp-1* germline.

## Description

In the *C. elegans* germline, GLP-1 Notch signaling promotes the stem cell fate and its loss results in a germline proliferation defective phenotype (Glp) where, on average, the single germ cell that corresponds to the progenitor for each gonad arm undergoes 2 rounds of division before prematurely entering into meiosis, and differentiating into sperm (Austin and Kimble, 1987). Downstream of GLP-1 Notch signaling, GLD-1 and GLD-2 pathways (GLD) redundantly promote germ cell meiotic entry. GLP-1 Notch signaling represses both pathways to promote the stem cell fate (Kadyk and Kimble, 1998; Hansen *et al.*, 2004). GLD pathway single mutants exhibit essentially normal meiotic entry. A simultaneous disruption of genes from both the GLD-1 and the GLD-2 pathways results in a tumorous germline phenotype due to a defect in meiotic entry. The tumorous phenotype of GLD double mutants is epistatic to *glp-1(-),* consistent with GLP-1 signaling promoting the stem cell fate by inhibiting the redundant GLD pathways. In *gld-1(-); glp-1(-),* the germline is Glp, however the single germ cell undergoes 2-3 additional rounds of division before entering into meiosis, as compared to *glp-1(-)* (Kadyk and Kimble, 1998; Francis *et al.*, 1995). We therefore expected a similar Glp phenotype in the mutant germlines of *gld-2* pathway genes, namely *gld-2* and *gld-3*, when GLP-1 signaling is compromised. Here, we examined dissected young adult (8-hr post-adult molt) hermaphrodite germlines by REC-8 (progenitor zone marker), HIM-3 (meiotic prophase marker) and MSP (sperm/spermatogenesis marker). As expected, we found that germlines of both genotypes were Glp (absence of REC-8 positive nuclei and presence of HIM-3 positive nuclei). Compared with *glp-1(-)* germlines, the total number of germ cells was slightly higher in *gld-2(-); glp-1(-)* germlines (Fig 1)(Kadyk and Kimble, 1998). In contrast, the total number of germ cells was significantly higher, and variable, in *gld-3(-); glp-1(-)* double mutant germlines with some of the gonads having more than 100 germ cells (Fig 1). Thus, like GLD-1, both GLD-2 and GLD-3 appear to repress the stem cell fate in the absence of GLP-1 signaling, although the mechanisms are unknown. That loss of GLD-3 results in much higher number of germ cells compared to GLD-2, once again underlines the functional non-equivalency of GLD-2 and GLD-3 (see Mohammad *et al.*, 2018). One possibility is that GLD-4 partially compensates for GLD-2 loss, thus keeping the number of germ cells lower in *gld-2(-); glp-1(-)* germlines (Millonigg *et al.*, 2014). It will be of interest to analyze at what point during larval development the *gld-3(-); glp-1(-)* germlines lose the stem cell pool.

## Methods

Hermaphrodites were grown on NGM plates with OP50 bacteria at 20^o^. Young adults, eight-hours after the L4/adult molt, were dissected and stained with anti-HIM-3 (rabbit, 1:100) (Zetka *et al.*1999), anti-REC-8 (rat, 1:100) (Pasierbek *et al.*, 2001) and anti-MSP (mouse,1:10,000) (Kosinski *et al.* 2005) antibodies along with DAPI and imaged as described (Mohammad *et al.*, 2018). Z-stacked images were further processed in Fiji and assembled in Inkscape. The nuclei counting was carried out in Fiji and further data processing and plotting was done using Python, Awk and R. The following strains were used. BS3854: *+ I/hT2 (I;III); glp-1(q175) III/hT2 [bli-4(e937) let-?(q782) qIs48] (I;III).*BS4253: *gld-2(q497) I/hT2 (I;III); glp-1(q175) III/hT2 [bli-4(e937) let-?(q782) qIs48] (I;III).*BS4259: *+ I/hT2 (I;III); gld-3(q730)/mIn1 [mIs14 dpy-10(e128)] II; glp-1(q175) III/hT2 [bli-4(e937) let-?(q782) qIs48] (I;III).*The alleles used for *glp-1*, *gld-2* and *gld-3* containing strains are the canonical null alleles (Austin and Kimble, 1987; Kadyk and Kimble, 1998; Francis *et al.*, 1995). Strains are available with the authors upon request.

## References

[R1] Austin J, Kimble J (1987). glp-1 is required in the germ line for regulation of the decision between mitosis and meiosis in C. elegans.. Cell.

[R2] Francis R, Maine E, Schedl T (1995). Analysis of the multiple roles of gld-1 in germline development: interactions with the sex determination cascade and the glp-1 signaling pathway.. Genetics.

[R3] Hansen D, Hubbard EJ, Schedl T (2004). Multi-pathway control of the proliferation versus meiotic development decision in the Caenorhabditis elegans germline.. Dev Biol.

[R4] Kadyk LC, Kimble J (1998). Genetic regulation of entry into meiosis in Caenorhabditis elegans.. Development.

[R5] Kosinski M, McDonald K, Schwartz J, Yamamoto I, Greenstein D (2005). C. elegans sperm bud vesicles to deliver a meiotic maturation signal to distant oocytes.. Development.

[R6] Millonigg S, Minasaki R, Nousch M, Novak J, Eckmann CR (2014). GLD-4-mediated translational activation regulates the size of the proliferative germ cell pool in the adult C. elegans germ line.. PLoS Genet.

[R7] Mohammad A, Vanden Broek K, Wang C, Daryabeigi A, Jantsch V, Hansen D, Schedl T (2018). Initiation of Meiotic Development Is Controlled by Three Post-transcriptional Pathways in *Caenorhabditis elegans*.. Genetics.

[R8] Pasierbek P, Jantsch M, Melcher M, Schleiffer A, Schweizer D, Loidl J (2001). A Caenorhabditis elegans cohesion protein with functions in meiotic chromosome pairing and disjunction.. Genes Dev.

[R9] Zetka MC, Kawasaki I, Strome S, Müller F (1999). Synapsis and chiasma formation in Caenorhabditis elegans require HIM-3, a meiotic chromosome core component that functions in chromosome segregation.. Genes Dev.

